# Self-Assembly of Nanovoids in Si Microcrystals Epitaxially Grown on Deeply Patterned Substrates

**DOI:** 10.1021/acs.cgd.9b01312

**Published:** 2020-04-08

**Authors:** Andrea Barzaghi, Saleh Firoozabadi, Marco Salvalaglio, Roberto Bergamaschini, Andrea Ballabio, Andreas Beyer, Marco Albani, Joao Valente, Axel Voigt, Douglas J. Paul, Leo Miglio, Francesco Montalenti, Kerstin Volz, Giovanni Isella

**Affiliations:** † L-NESS, Dipartimento di Fisica, Politecnico di Milano, Via Anzani 42, 22100 Como, Italy; ‡ Materials Science Center and Faculty of Physics, Philipps-Universität Marburg, Hans-Meerweinstraße 6, 35032 Marburg, Germany; § Institute of Scientific Computing, Technische Universität Dresden, 01062 Dresden, Germany; ∥ Dresden Center for Computational Materials Science, Technische Universität Dresden, 01062 Dresden, Germany; ⊥ L-NESS and Dipartimento di Scienza dei Materiali, Università di Milano-Bicocca, Via R. Cozzi 55, I-20125 Milano, Italy; # James Watt School of Engineering, University of Glasgow, Rankine Building, Oakfield Avenue, Glasgow G12 8LT, United Kingdom

## Abstract

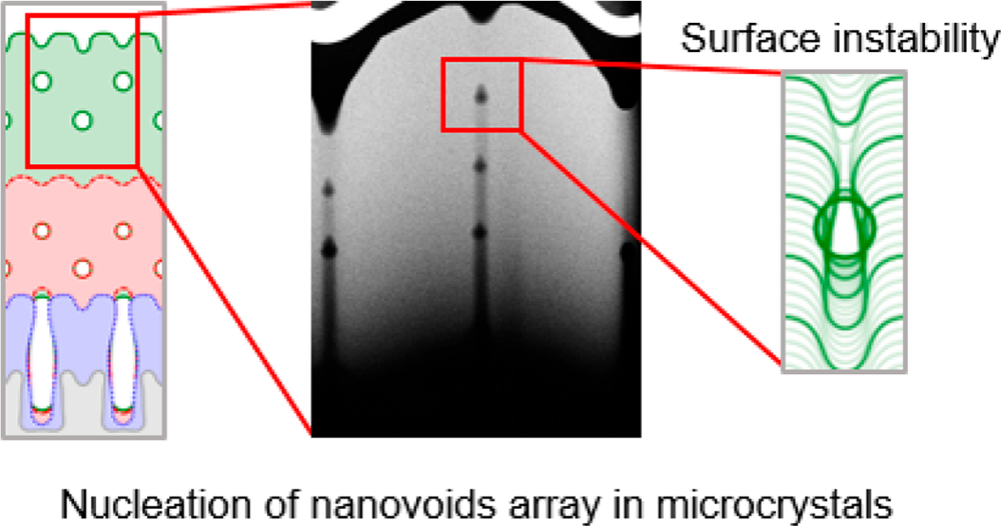

We present an experimental and theoretical analysis of the formation of nanovoids within Si microcrystals epitaxially grown on Si patterned substrates. The growth conditions leading to the nucleation of nanovoids have been highlighted, and the roles played by the deposition rate, substrate temperature, and substrate pattern geometry are identified. By combining various scanning and transmission electron microscopy techniques, it has been possible to link the appearance pits of a few hundred nanometer width at the microcrystal surface with the formation of nanovoids within the crystal volume. A phase-field model, including surface diffusion and the flux of incoming material with shadowing effects, reproduces the qualitative features of the nanovoid formation thereby opening new perspectives for the bottom-up fabrication of 3D semiconductors microstructures.

## Abstract 2

Ordered 3D arrays of nanovoids can be formed during the epitaxial growth of Si microcrystals on patterned substrates. Phase-field simulations provide a clear link between nanovoid formation and the ratio between diffusion coefficient and deposition rate. With the appropriate combination of pattern geometry, deposition rate and deposition temperature control over nanovoids formation could be achieved also with other semiconductors.

## Introduction

In recent years the monolithic integration of group IV and III–V semiconductors on silicon has been widely investigated as a viable pathway to go beyond Moore’s law. This approach needs to address the challenges inherent to heteroepitaxy, which are mostly stemming from the lattice and thermal expansion coefficient mismatch between the substrate and the epilayer.

A novel approach named vertical heteroepitaxy (VHE), which combines epitaxial growth and substrate patterning, has been shown to substantially mitigate these issues.^1^ Under strong out-of-equilibrium growth conditions, obtained by combining high deposition rates and relatively low growth temperatures, the epitaxial deposition of Ge on deeply patterned Si substrates results in the vertical growth of an array of Ge microcrystals, which can be separated by tens of nanometers gap^2^ or eventually merge to form a suspended layer.^3,4^

The material quality of such microcrystals has been deeply investigated, showing that the thermal strain is indeed fully relaxed^5^ and that all the threading dislocations can be expelled from the crystals.^6^ Moreover, it has been predicted^7^ and experimentally verified^8,9^ that, by decreasing the size of the pillars etched into the substrate and by linearly grading the compositional profile, it is possible to achieve full elastic relaxation without the nucleation of misfit dislocations.

Tuning the morphology of the crystals obtained by VHE is crucial to exploit the aforementioned properties in a controlled fashion. Several aspects of the morphological evolution of the microcrystals, such as the onset of vertical growth, the different faceting due to growth conditions, and the dynamics of merging have already been investigated both by experiments and theory.^1,2,4^ Some features originating from the unique combination of deep substrate patterning and high growth rates typical of VHE still need to be addressed and understood in detail by dedicated experiments and theoretical models.

In this work, we focus on the formation of self-assembled nanovoids arranged in ordered arrays, formed during VHE, within each microcrystal and in between merging microcrystals. We consider a prototypical system made of Si microcrystals grown on Si pillars, allowing us to focus on the main physical aspects of the growth and avoid the additional complexity of heteroepitaxy. The growth conditions leading to the nucleation of ordered arrays of nanovoids are highlighted and the role played by growth parameters, such as deposition rate and temperature, is clarified by a theoretical analysis involving a continuum model and simulations of the material deposition on nonflat substrates.

This study sheds light on the self-assembly formation of nanometric voids in microcrystals and sets the ground for control of the voids. It is worth mentioning that highly controllable arrays of voids in silicon have already been obtained by etching deep via holes and subsequently annealing the sample.^10,11^ The spontaneous formation of voids during epitaxial growth of 3D crystals, however, which significantly differs from conventional planar configurations, has not been observed and investigated yet. This opens new perspectives in exploiting the bottom-up fabrication of 3D, semiconductor microstructures with potential applications in the fabrication of silicon-on-nothing,^12^ MEMS, and photonic crystal^13^ devices. The possibility of combining different semiconducting material adds an additional degree of freedom in the fabrication of 3D photonic crystals. The operating wavelength could be extended in the mid-infrared by exploiting the higher transparency, in this wavelength range, of germanium as compared to silicon. In addition, the modulation of the refractive index due to void formation could be combined with that arising from the alloying of different semiconductors, making the SiGe system a relevant candidate.^14^

The presence of additional free surfaces within the Si microcrystals may also be exploited to modify the elastic and plastic properties of the microcrystals.

## Methods

Patterned substrates have been fabricated by dry-etching square Si pillars on a Si (001) wafer. The typical etching depths were 10 and 2.7 μm depending on the technique, optical or electron-beam lithography, used for the pattern transfer. Each substrate features several regions, each one characterized by a given pillar width *W* and separating gap *G* with dimensions varied between 1 to 4 μm.

Before epitaxial growth, performed in a low-energy plasma-enhanced CVD (LEPECVD) reactor, the patterned substrates were cleaned by RCA, followed by an HF dip for oxide removal. LEPECVD exploits a low energy and high-density argon plasma to efficiently decompose the gas phase precursors,^15^ resulting in a deposition rate of ∼5 nm/s almost independent of the substrate temperatures, which is 700 °C for the samples analyzed in this work (if not stated otherwise). The combination of high rate and low deposition temperature leads to a strong out-of-equilibrium deposition process where kinetic effects dominate over thermodynamic effects. This is a key feature of LEPECVD. Indeed, deposition processes operating closer to thermodynamic equilibrium, such as thermal CVD, do not result in the vertical growth of microcrystals.^16^ A rough estimate of the adatoms diffusion length *L*_d_, achievable by LEPECVD, can be obtained by assuming that surface diffusion dominates over bulk diffusion and, consequently, considering the average surface diffusion time equal to the time required for the deposition of a monolayer, i.e. inversely proportional to the growth rate. By taking typical values for the diffusion coefficient in Si homoepitaxy from the literature,^17^ it is possible to estimate *L*_d_ to be comprised between a few hundred nanometers and a few micrometers. As explained in detail in ref (2), control over microcrystal morphology can be achieved only for diffusion lengths comparable with the Si pillar size; therefore, in this work growth parameters have been set to achieve *L*_d_ ≈ 1 μm and the Si pillar size varied in the micrometre range.

The serial-sectioning technique has been applied to characterize the microcrystal morphology in three dimensions. A focused ion beam/scanning electron microscope (FIB/SEM) dual beam tool has been used for slicing the microcrystals using Ga ions and subsequently imaging each section using secondary electrons in SEM (JEOL JIB 4610F).^18−20^ With the help of the Avizo software package, the series of image slices have been reconstructed to a three-dimensional volume. Furthermore, using FIB/SEM, thin electron transparent lamellae have been prepared by cutting the microcrystals right at the center along the [110] direction.^21^ A double Cs-corrected scanning transmission electron microscope (JEOL 2200FS) operating at 200 kV along with an annular dark field detector (JEOL EM-24590YPDFI) has been used to image the ordered arrays of microvoids.

To analyze the experimental results a minimal, continuum two-dimensional model tackling the evolution of surfaces and encoding the main contributions to surface diffusion and the growth of vertical crystals^2^ has been implemented. In particular, we focus on the impinging material flux on nonflat surfaces, which is unevenly distributed due to self-shielding effects,^22^ and the material redistribution along the surface due to surface diffusion.^23^ Namely, we aim to describe the evolution of the surface of the solid phase by means of its normal velocity
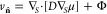
1with μ as the local chemical potential at the surface, proportional to the local curvature for isotropic surface energies, and ∇*_S_* as the surface Laplacian. *D* is the diffusion coefficient, which can be assumed to depend on the temperature *T* by an Arrhenius law *D* ∝ *A* exp(−*B*/*kT*) with *A* and *B* as positive constants. Φ corresponds to the (local) growth rate due to material deposition. Together with the temperature *T*, the magnitude of Φ can be controlled in the experiments, while its distribution at the surface of the solid phase generally depends on the deposition techniques, material anisotropies, and the geometry of the growing crystals through shielding effects.

In order to cope with topological changes as the formation of voids, we consider the implicit description of evolving surfaces achieved by the phase-field (PF) model introduced in refs (24 and 25) reproducing the dynamics encoded in eq 1.^26^ The phase-field function φ is set equal to 1 within the crystal and 0 outside and has a continuous transition in between, which is well-described by
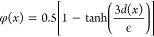
2with *d*(*x*) as the signed distance from the surface, namely the isosurface φ = 0.5, and ϵ as the thickness of the interface between the phases. The evolution law for φ reads
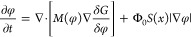
3Equation 3 approximates well the dynamics described by eq 1. The first term at the right-hand side of eq 3 encodes surface diffusion, where the surface free energy, *G*, is the Ginzburg–Landau energy functional,
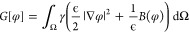
4where *B*(φ) = 18φ^2^(1 – φ)^2^ and *M*(φ) = (2*D*/ϵ)*B*(φ) is a mobility function with *D* as the diffusion coefficient defined above. The γ parameter accounts for the surface energy density, here assumed to be isotropic for the sake of simplicity. Anisotropy in γ^27^ is indeed expected to play a minor role in the dynamics of void formation with respect to the overall tendency toward surface smoothing enforced by local curvatures. This looks reasonable when comparing the isotropic evolution in ref (3) to the one in ref (28) where surface anisotropy was included to obtain the faceted shapes of the microcrystals. Additional effects, stemming from facet-dependent adatom kinetics,^29^ should also be considered to the best fit of the actual experimental morphologies,^2,30^ which is beyond the scope of the present study. The second term at the right-hand side of eq 3 stands for the microcrystal growth due to the material flux impinging at the surface and reproduces the contribution of Φ as in eq 1. Φ_0_ is a scaling factor taking into account the amount of incoming material. Here it is assumed to be isotropic. The function *S*(*x*) accounts for shielding effects, and it is computed by a Ray–Tracing algorithm. It is zero at a point ***x*** of the surface completely shielded with respect to the incoming material flux and 1 where shielding effects are not present as for a flat surface. In this way, on a flat surface, the growth rate is Φ_0_ everywhere; i.e., it corresponds to the nominal deposition flux. The competition between the two terms in eq 3 is controlled by the ratio *D*/Φ_0_. Different values for *D* and Φ_0_ with same values for this ratio would indeed provide the same morphological evolution, just occurring on a different time scale. We then fix Φ_0_ = 1, so that this ratio is directly controlled by *D* without loss of generality. Time and length scales directly entering eqs 2–4 are given in dimensionless units. Indicatively, large values of *D*/Φ_0_ correspond to high temperatures and low deposition rates, while small *D*/Φ_0_ values reproduce low temperatures and high deposition rates. The simulations reported in the following are performed by using the finite element toolbox AMDiS.^31,32^ Further details about this specific PF model and the Ray–Tracing procedure employed to compute *S*(*x*) can be found in ref (26). Details of this theoretical approach are illustrated in Figure 4a.

## Results and Discussion

Microcrystals grown on deeply etched Si substrates exhibit clear crystallographic facets with well-defined orientations, corresponding to the most stable crystal planes of the Si face centered cubic (FCC) crystal, i.e. (001), {111}, and {113}. Their relative dimensions, and therefore the final crystal shape, are however determined by kinetic parameters, i.e. by the relative growth rates of the facets.^2^ These are influenced by the diffusion lengths of the adatoms on each facet, which, in turn, depend on both the deposition temperature and rate.

**Figure 1:**
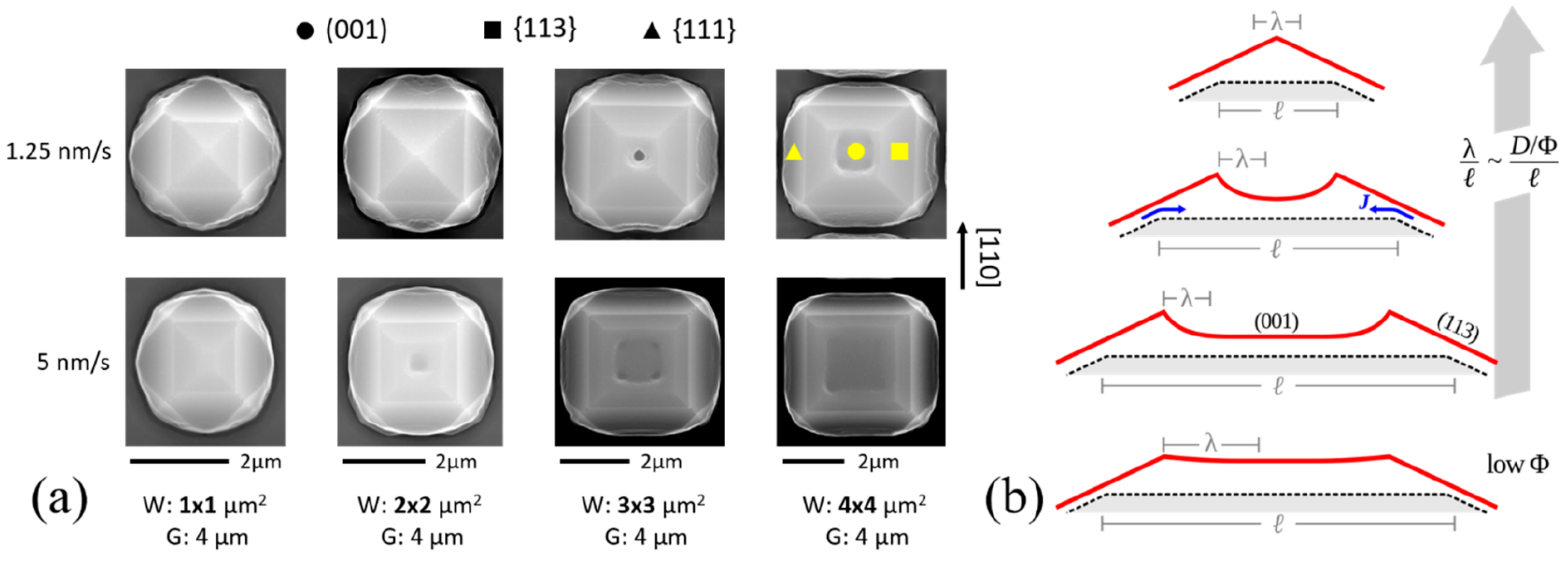
(a) Morphological evolution as a function of patterning (pillar size *W* and gap *G*) and deposition rate in 5 μm tall Si microcrystals, grown at 700 °C. (b) Schematic representation of the variation in the top morphology as a function of the size *l* of the (001) top facet (for a given diffusion length λ) determined by the material transfer *J* from the (113) to the (001) facet. The effect of a lower growth rate Φ, i.e. of longer λ, is also sketched.

This is outlined in Figure 1a, where 5 μm tall microcrystals deposited at 700 °C at two different growth rates (1.25 and 5 nm/s) are compared for patterned substrates with pillar width *W* varying between 1 and 4 μm and a separating gap *G* = 4 μm. In the case of low deposition rate and *W* = 1 and *W* = 2 μm, only {113} (at the center) and {111} facets can be observed. As the pillar size increases, the flat (001) surface appears at the top of the crystals. In the high deposition rate case, the (001) facet can also be found on top of pillars smaller than 3 × 3 μm^2^ and occupies a larger fraction of the microcrystal surface as compared to the corresponding sample grown at a lower rate.

The observed trend in the morphological evolution appears to be well consistent with the general picture of the growth process discussed for Ge/Si in ref (2). Indeed, also in the case of Si/Si microcrystals, {113} facets are found to grow slower than the (001) top facet. This is explained by considering a transfer of material from {113} facets to the (001) facet, due to their different incorporation rates.^29^ As illustrated in Figure 1b, such material spreads over a distance of the order of the diffusion length λ. As expected, λ increases when the diffusion coefficient, *D*, is increased i.e. with temperature, while it decreases for increasing deposition rates, since adatoms are more rapidly incorporated into the growing crystal for a larger incoming flux Φ_0_. The resulting morphology then depends on the ratio between λ and the (001) facet dimension *l*, which, in turn, is proportional to the pillar base width *W* in the initial growth stages, as illustrated in the Figure 1b. When λ ≳ *l*/2, the adatom flow spreads quite uniformly over the whole (001) surface, provoking an enhancement of its growth rate and leading, eventually, to a pyramidal shape dominated by the low growth rate {113} facets as predicted by the Borgstrom facet construction for a convex microstructure. On the contrary, if λ ≪ *l*/2 adatoms tend to accumulate at the edges of the (001) facet, then this results in the formation of ridges along the (001) perimeter. When the (001) region shrinks to 2λ, the mounds get close enough to overlap and form a concave region. Such pits are indeed evident in the top views of Figure 1a. A single pit is observed at the crystal center in the case of *W* = 3 μm and low rate, while four distinct pits are visible at the corners of the (001) facet in the case of *W* = 4 μm and low rate or *W* = 3 and 4 μm and at a high rate.

**Figure 2:**
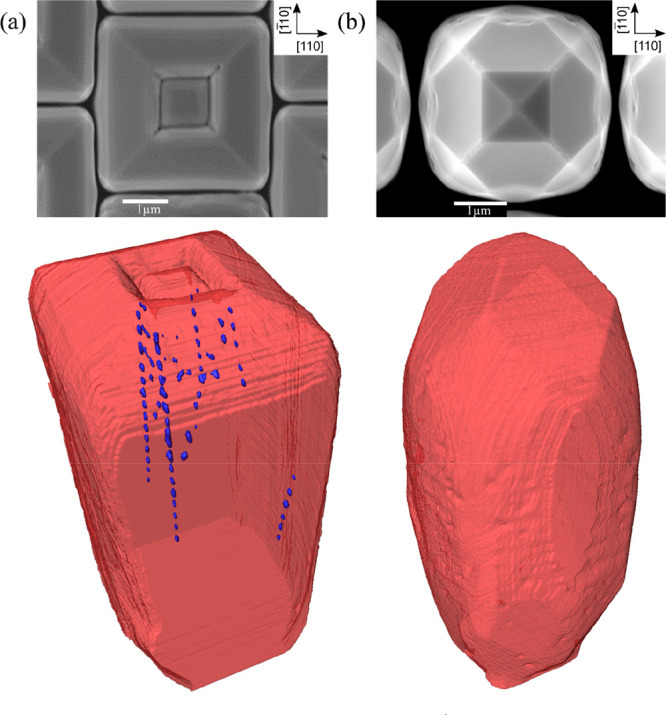
A SEM top view and 3D reconstruction obtained from FIB/SEM cross sections of 5 μm tall silicon microcrystals where (a) 4 pits are visible at the top (*W* = 2 × 2 μm^2^, *G* = 2 μm, and growth rate 1.25 nm/s) and (b) no pits can be observed (*W* = 1 × 1 μm^2^, *G* = 1 μm, and growth rate 4 nm/s).

To better analyze the origin of such surface pits, crystals showing both four pits and no pits have been characterized by FIB/SEM tomography, shown in Figure 2 together with the SEM top views. In the case of the samples featuring four pits (Figure 2a), arrays of small voids can be found inside the crystal beneath the four pits observed in the top view image. The four lines of voids are neither perfectly regular nor vertical, due to the evolution of the (001) facet during the deposition process. In the case of microcrystals terminated by {113} facets (Figure 2b), no voids can be seen in the tomography.

**Figure 3:**
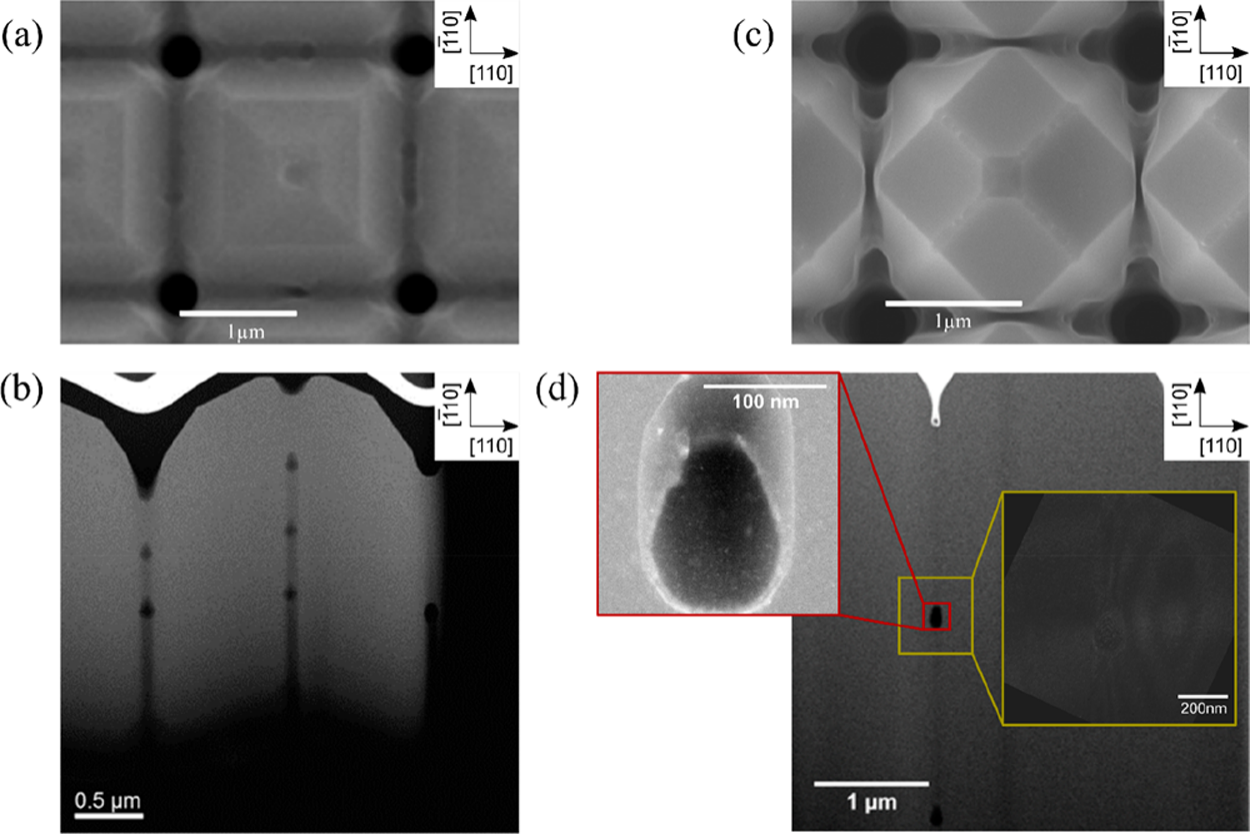
TEM cross sections of 5 μm tall Si microcrystals, grown at 720 °C and partially merged with the neighboring ones. The crystals are cut along the red lines in the SEM images during the TEM lamella preparation. (a) Microcrystals grown at a rate of 5 nm/s where a single pit is visible in the top view SEM image. (b) Two separate arrays of regularly spaced voids are visible, one at the center of the crystals and one in the merging region. (c) Merged microcrystals grown at a rate of 1.25 nm/s where no pit is visible in the top view SEM image. (d) TEM cross section of one nanovoid in the merging region between two crystals grown at 1.25 nm/s. The STEM LAADF image (red inset) shows well-defined facets, while no dislocations are observed near the void in the STEM WBDF image (yellow inset).

Microcrystals with a single pit at the center of the top surface have been investigated by annular dark-field scanning transmission electron microscopy (ADF-STEM) in samples where the crystals are partially merged with their neighbors (Figure 3). The lamellae have been obtained by FIB cutting the microcrystals through their center along the [110] direction, as indicated by the red lines also shown in Figure 3. Two perfectly regular sets of ∼100 nm large voids can be observed in Figure 3b: one beneath the central pit, and one in the merged region between the two crystals. The spacing between the voids is ∼450 nm and ∼350 nm, respectively. A comparison between the SEM top view (Figure 3a) and the TEM cross section (Figure 3b) indicates that the voids within the microcrystals are placed right at the center of each microcrystal, while those in the merging region might actually be displaced toward the microcrystal corner. The red inset in Figure 3d shows a low-angled ADF-STEM image of a single void, which is bounded by well-defined crystalline facets, in particular the {111} and (001) ones. Moreover, a transmission electron microscopy weak beam dark field (TEM-WBDF) image (yellow block in Figure 3b) proves that no dislocations are generated at the interface with the voids, or in the merged region.

The phase-field model illustrated in the previous section accurately reproduces the electron microscopy observations. In the low-diffusion regimes, profiles similar to those found at the top of the microcrystals are known to be unstable against the so-called shadowing instability:^22,33,34^ the bottom of the pit collects less material than their lateral ridges, which then grow and eventually merge, thus forming a void below the merging point.^26^ As discussed first in refs (1 and 2), the deposition technique considered here ensures short diffusion lengths and together with shielding effects enables vertical growth. The presence of voids may then originate from the pits observed at the top of the Si crystals, which are present since the early stages of growth in the surface profile, possibly because of the mechanism illustrated in Figure 1b.

In order to assess the role played by deposition with shadowing effects, surface diffusion, and the geometry of the evolving crystals, phase-field simulations are performed to analyze the formation of such nanovoids in the prototypical case of a single pit at the center of the crystal top facet, to mimic the microcrystals grown at a rate of 5 nm/s in Figure 3a and 3b.

**Figure 4:**
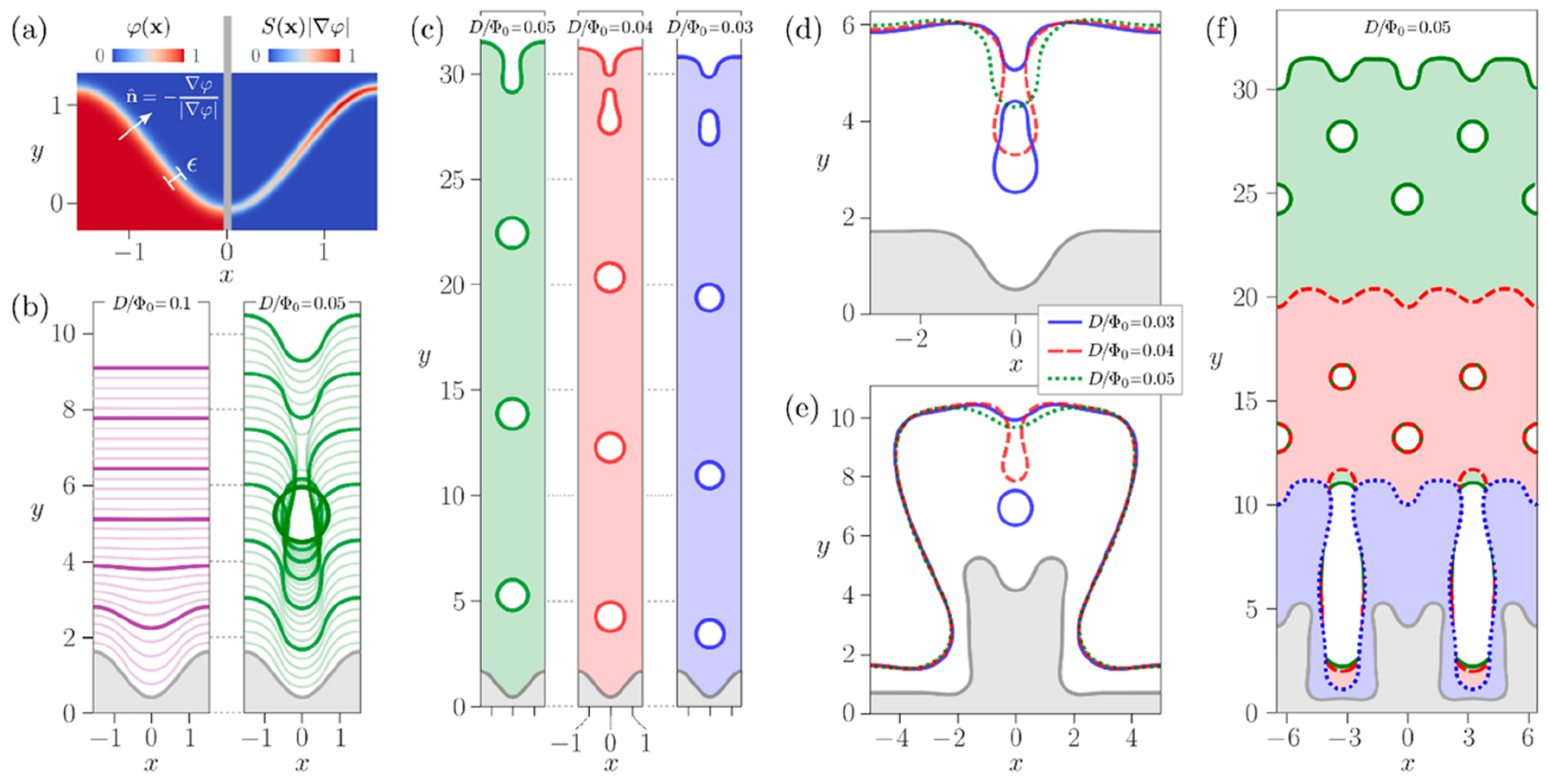
PF simulations of growth and formation of voids due to the deposition with shadowing effects on nonflat surfaces. (a) Details of the PF model: diffuse interface representation of a sinusoidal surface profile by means of φ(*x*) (left) and S(*x*) for isotropic material deposition (right). Surface profiles reported in the following panels correspond to the isosurface φ = 0.5. Gray areas correspond to initial profiles. (b) Sequence of profiles (at intervals Δ*t* during the deposition on a sinusoidal surface with *D*/Φ_0_ = 0.1 (left) and *D*/Φ_0_ = 0.05 (right), Δ*t* = 0.25, *t*_tot_ = 7.5), respectively. (c) Comparison between morphologies and arrays of voids obtained by deposition on a profile as in (b) with different values of *D*/Φ_0_ at *t* = 25. (d) A comparison of the morphologies after the deposition on a pit connected to flat regions, *t* = 4.3. (e) A comparison of the morphologies after the deposition on a pit embedded in a vertical structure, *t* = 5. (f) The formation of voids at the center and between growing vertical structures shown by three profiles during the deposition on structures as in panel (e) with a smaller gap in between.

We focus first on a periodic surface profile *p*(*x*) = *Acos*(*Lx*), mimicking the shape of a pit formed at the center of the pillar top (or, similarly, the pit formed at the coalescence point between neighboring crystals). Figure 4a shows the profile obtained for ϵ = π/20, *A* = π/5, and *L* = π by means of φ(*x*) (left) and *S*(*x*) |∇φ| (right). When surface diffusion is the dominant mechanism during growth, i.e. for relatively large diffusion coefficients, this corrugation of the surface is expected to vanish during growth.^26^ This is demonstrated in Figure 4b where *D*/Φ_0_ = 0.1. By decreasing *D*/Φ_0_ below a certain threshold, the shadowing instability sets in. This is shown in Figure 4b where *D*/Φ_0_ = 0.05 and vertical structures form at the peaks of the initial surface profile. Then, these ridges extend along the in-plane direction due to the combined effect of material collected from the external flux and the material redistribution by surface diffusion. As a result, the ridges merge and a buried void in the solid phase is formed. The surface profile remains corrugated after the merging. The resulting shape may still be unstable against the shadowing instability leading to the growth of additional vertical structures with the formation of additional voids (see the growing vertical structures in Figure 4b, right) arranged into an ordered array as in Figure 4c for *D*/Φ_0_ = 0.05.

These simulations qualitatively account for the formation of nanovoids ascribing it to the combination of self-shielding of material flux, a limited contribution of surface diffusion, and the initial corrugated surface. By decreasing *D*/Φ_0_ further, the material redistribution is limited to shorter distances. This leads to a larger accumulation of material close to the top regions and then to a faster formation of nanovoids. This results in a controllable distribution of voids as illustrated in Figure 4c, where the number of voids per unit length is found to increase by decreasing *D*/Φ_0_ (the opposite limit is no void formation for a large *D*/Φ_0_). We recall that the diffusion coefficient is expected to follow the Arrhenius law.^23^ Therefore, the changes in the distribution of voids illustrated in Figure 4c by increasing/decreasing *D*/Φ_0_ can be achieved in experiments by raising/diminishing the growth temperature. Analogously, according to the definition of parameters (see eq 2 and ref (26)), these trends can be obtained by varying the growth rate in the opposite way. This is actually what is observed by the experiments discussed in Figure 1, where voids form by increasing the growth rate. The limit for vanishing contribution of the surface diffusion, i.e. for very small *D*/Φ_0_ values or very low temperatures and/or very high deposition rates, is a dendritic growth of the crystals.^22,33,34^ It is worth mentioning, however, that the theoretical approach adopted here can only describe features larger than ϵ and therefore this regime cannot be explored.

The simulations discussed so far focus on an idealized, periodic surface profile. The array of voids as observed in the experiments, however, forms at the center of a large microcrystal. In order to prove that the shadowing instability may become established also for profiles which better resemble experimental systems, we consider two limiting cases: a pit connected to an extended flat surface (Figure 4d), mimicking the effect of having a single small perturbation at the center of a large crystal, and a pit embedded in a vertical structure (Figure 4e), accounting for the presence of lateral surfaces. In both cases we consider a pit-like morphology corresponding to a period of the periodic perturbation *p*(*x*). As shown by the comparison at similar times of surface profiles obtained with different values of *D*/Φ_0_, the same phenomenology in terms of shadowing instability and hierarchy in the formation of voids when varying *D*/Φ_0_ is reproduced. As both curvature and material flux distribution at the surface are different with respect to the periodic profile, different quantitative features are expected, but the detailed analysis of these features is beyond the present work. From Figure 4e one can also notice that the range of *D*/Φ_0_ explored is sufficient to observe differences concerning voids formation at the center, but it does not significantly affect the global shape of the growing structure, even in this simplified simulation where the relative size of the pit is larger than in the experiments. This further confirms the unstable nature of the underlying process for voids formation. Moreover, it points out the different length scales at which the growth of the vertical crystals and the formation of voids may occur.

The configuration illustrated in Figure 4e allows us to consider also the formation of voids together with the coalescence of neighboring crystals as lateral growth would eventually lead to their merging. This is illustrated in Figure 4f. The spacing between crystals is set here to be as large as the initial vertical structure and *D*/Φ_0_ = 0.05. In this system, along with the initial pit at the center of the crystal triggering the formation of a void at the center, a similar pit forms over the merged region as is also reported in previous works.^3,4^ This eventually triggers a similar instability mechanism and leads to the formation of an additional void array aligned with the trenches. At later stages, as observed for the more idealized profiles of Figure 4f, the mechanism repeats in both regions with the formation of voids aligned with the center and with the trenches of the patterned substrate. This evolution qualitatively reproduces the evidence illustrated in Figure 3b, thus further assessing the origin of voids formation and their alignment in the different portions of the crystal.

The theoretical investigation performed with the aid of phase-field simulations focused here on explaining the phenomenology observed in the experiments and understanding the main mechanisms at play. An interesting perspective consists of performing a systematic analysis of geometries together with an extended set of simulations, thus providing a full overview of morphological changes in terms of the physical parameters entering the model (e.g., T, D, and Φ_0_). A response diagram concerning the phenomenology illustrated in Figure 4b is already reported in ref (26) and can be readily exploited to frame both theoretical and experimental results from a qualitative point of view. Quantitative prediction would require instead extended analysis of geometries closely resembling the experimental system, thus including an extended set of parameters to be investigated. Moreover, phase-field models accounting for anisotropies and three-dimensional simulations should be also considered. Such extensions will be explored in future studies.

## Conclusions

We have demonstrated that ordered 3D arrays of nanovoids can be formed during the epitaxial growth of Si microcrystals on Si patterned substrates. SEM and TEM analysis demonstrate that, in correspondence to pits developing on the microcrystal surface, arrays of nanovoids are formed within the microcrystal. The ratio between the pattern size and the diffusion length has been identified as the key parameter for the onset of a shadowing instability which gives rise to surface pits and, eventually, to nanovoid formation. Phase-field simulations, taking into account surface diffusion, the flux of incoming material, and shadowing provide a clear link between nanovoid formation and the ratio between the diffusion coefficient and the deposition rate. Therefore, with the appropriate combination of pattern geometry, deposition rate and deposition temperature control over nanovoids formation could be achieved also with deposition techniques different from LEPECVD. By tuning the substrate patterning and deposition parameters, it should be possible to exploit this technique to fabricate self-assembled arrays of voids with controllable dimensions and spacing. The technique could also be extended to other semiconducting materials such as germanium and find application in the fabrication of 3D photonic crystals.

## References

[ref1] Falub C. V.; von Känel H.; Isa F.; Bergamaschini R.; Marzegalli A.; Chrastina D.; Isella G.; Muller E.; Niedermann P.; Miglio L. Scaling Hetero-Epitaxy from Layers to Three-Dimensional Crystals. Science (Washington, DC, U. S.) 2012, 335, 1330–1334. 10.1126/science.1217666. 22422978

[ref2] Bergamaschini R.; Isa F.; Falub C. V.; Niedermann P.; Müller E.; Isella G.; Von Känel H.; Miglio L. Self-Aligned Ge and SiGe Three-Dimensional Epitaxy on Dense Si Pillar Arrays. Surf. Sci. Rep. 2013, 68, 390–417. 10.1016/j.surfrep.2013.10.002.

[ref3] Salvalaglio M.; Bergamaschini R.; Isa F.; Scaccabarozzi A.; Isella G.; Backofen R.; Voigt A.; Montalenti F.; Capellini G.; Schroeder T.; Von Känel H.; Miglio L. Engineered Coalescence by Annealing 3D Ge Microstructures into High-Quality Suspended Layers on Si. ACS Appl. Mater. Interfaces 2015, 7, 19219 10.1021/acsami.5b05054. 26252761

[ref4] Bergamaschini R.; Salvalaglio M.; Scaccabarozzi A.; Isa F.; Falub C. V.; Isella G.; Von Känel H.; Montalenti F.; Miglio L. Temperature-Controlled Coalescence during the Growth of Ge Crystals on Deeply Patterned Si Substrates. J. Cryst. Growth 2016, 440, 86–95. 10.1016/j.jcrysgro.2016.01.035.

[ref5] Chrastina D.; Kreiliger T.; Isella G.; Falub C. V.; von Känel H.; Dommann A.; Taboada A. G.; Marzegalli A.; Meduňa M.; Isa F.; Miglio L. Perfect Crystals Grown from Imperfect Interfaces. Sci. Rep. 2013, 3, 1–6. 10.1038/srep02276. PMC3721082 23880632

[ref6] Marzegalli A.; Isa F.; Groiss H.; Müller E.; Falub C. V.; Taboada A. G.; Niedermann P.; Isella G.; Schäffler F.; Montalenti F.; Von Känel H.; Miglio L. Unexpected Dominance of Vertical Dislocations in High-Misfit Ge/Si(001) Films and Their Elimination by Deep Substrate Patterning. Adv. Mater. 2013, 25, 4408–4412. 10.1002/adma.201300550. 23788016

[ref7] Salvalaglio M.; Montalenti F. Fine Control of Plastic and Elastic Relaxation in Ge/Si Vertical Heterostructures. J. Appl. Phys. 2014, 116, 104306 10.1063/1.4895486.

[ref8] Montalenti F.; Salvalaglio M.; Marzegalli A.; Zaumseil P.; Capellini G.; Schülli T. U.; Schubert M. A.; Yamamoto Y.; Tillack B.; Schroeder T. Fully Coherent Growth of Ge on Free-Standing Si(001) Nanomesas. Phys. Rev. B: Condens. Matter Mater. Phys. 2014, 89, 1–7. 10.1103/PhysRevB.89.014101.

[ref9] Isa F.; Salvalaglio M.; Arroyo Y.; Dasilva R.; Meduna M.; Barget M.; Jung A.; Kreiliger T.; Isella G.; Erni R.; Pezzoli F.; Bonera E.; Niedermann P.; Gröning P.; Montalenti F. Highly Mismatched, Dislocation-Free SiGe/Si Heterostructures. Adv. Mater. 2016, 28, 884–888. 10.1002/adma.201504029. 26829168

[ref10] Sato T.; Mitsutake K.; Mizushima I.; Tsunashima Y. Micro-Structure Transformation of Silicon: A Newly Developed Transformation Technology for Patterning Silicon Surfaces Using the Surface Migration of Silicon Atoms by Hydrogen Annealing. Jpn. J. Appl. Phys. 2000, 39, 5033–5038. 10.1143/JJAP.39.5033.

[ref11] Mueller T.; Dantz D.; von Ammon W.; Virbulis J.; Bethers U. Modeling of Morphological Changes by Surface Diffusion in Silicon Trenches. ECS Transactions 2006, 363–374. 10.1149/1.2195673.

[ref12] Mizushima I.; Sato T.; Taniguchi S.; Tsunashima Y. Empty-Space-in-Silicon Technique for Fabricating a Silicon-on-Nothing Structure. Appl. Phys. Lett. 2000, 77, 3290–3292. 10.1063/1.1324987.

[ref13] Gupta S.; Tietz S.; Vuckovic J.; Saraswat K. Silicon-Compatible Fabrication of Inverse Woodpile Photonic Crystals with a Complete Band Gap. ACS Photonics 2019, 6, 368–373. 10.1021/acsphotonics.8b01000.

[ref14] Ramirez J. M.; Vakarin V.; Frigerio J.; Chaisakul P.; Chrastina D.; Le Roux X.; Ballabio A.; Vivien L.; Isella G.; Marris-Morini D. Ge-Rich Graded-Index Si_1-XGex Waveguides with Broadband Tight Mode Confinement and Flat Anomalous Dispersion for Nonlinear Mid-Infrared Photonics. Opt. Express 2017, 25, 6561 10.1364/OE.25.006561. 28381003

[ref15] Rosenblad C.; Deller H. R.; Dommann A.; Meyer T.; Schroeter P.; von Känel H. Silicon Epitaxy by Low-Energy Plasma Enhanced Chemical Vapor Deposition. J. Vac. Sci. Technol., A 1998, 16, 2785–2790. 10.1116/1.581422.

[ref16] Skibitzki O.; Capellini G.; Yamamoto Y.; Zaumseil P.; Schubert M. A.; Schroeder T.; Ballabio A.; Bergamaschini R.; Salvalaglio M.; Miglio L.; Montalenti F. Reduced-Pressure Chemical Vapor Deposition Growth of Isolated Ge Crystals and Suspended Layers on Micrometric Si Pillars. ACS Appl. Mater. Interfaces 2016, 8, 26374–26380. 10.1021/acsami.6b07694. 27603117

[ref17] Mo Y. W.; Kleiner J.; Webb M. B.; Lagally M. G. Surface Self-Diffusion of Si on Si(001). Surf. Sci. 1992, 268, 275–295. 10.1016/0039-6028(92)90968-C.

[ref18] Inkson B. J.; Mulvihill M.; Möbus G. 3D Determination of Grain Shape in a FeAl-Based Nanocomposite by 3D FIB Tomography. Scr. Mater. 2001, 45, 753–758. 10.1016/S1359-6462(01)01090-9.

[ref19] Kubis A. J.; Shiflet G. J.; Dunn D. N.; Hull R. Focused Ion-Beam Tomography. Metall. Mater. Trans. A 2004, 35, 1935–1943. 10.1007/s11661-004-0142-4.

[ref20] De Winter D. A. M.; Schneijdenberg C. T. W. M.; Lebbink M. N.; Lich B.; Verkleij A. J.; Drury M. R.; Humbel B. M. Tomography of Insulating Biological and Geological Materials Using Focused Ion Beam (FIB) Sectioning and Low-KV BSE Imaging. J. Microsc. 2009, 233, 372–383. 10.1111/j.1365-2818.2009.03139.x. 19250458

[ref21] Schaffer M.; Schaffer B.; Ramasse Q. Sample Preparation for Atomic-Resolution STEM at Low Voltages by FIB. Ultramicroscopy 2012, 114, 62–71. 10.1016/j.ultramic.2012.01.005. 22356790

[ref22] Bales G. S.; Zangwill A. Growth Dynamics of Sputter Deposition. Phys. Rev. Lett. 1989, 63, 692–692. 10.1103/PhysRevLett.63.692. 10041149

[ref23] Mullins W. W. Theory of Thermal Grooving. J. Appl. Phys. 1957, 28, 333–339. 10.1063/1.1722742.

[ref24] Li B.; Lowengrub J. Geometric Evolution Laws for Thin Crystalline Films: Modeling and Numerics. Commun. Comput. 2009, 433–482.

[ref25] Bergamaschini R.; Salvalaglio M.; Backofen R.; Voigt A.; Montalenti F. Continuum Modelling of Semiconductor Heteroepitaxy: An Applied Perspective. Adv. Phys. X 2016, 1, 331–367. 10.1080/23746149.2016.1181986.

[ref26] Salvalaglio M.; Backofen R.; Voigt A. Thin-Film Growth Dynamics with Shadowing Effects by a Phase-Field Approach. Phys. Rev. B: Condens. Matter Mater. Phys. 2016, 94, 30–32. 10.1103/PhysRevB.94.235432.

[ref27] Salvalaglio M.; Backofen R.; Bergamaschini R.; Montalenti F.; Voigt A. Faceting of Equilibrium and Metastable Nanostructures: A Phase-Field Model of Surface Diffusion Tackling Realistic Shapes. Cryst. Growth Des. 2015, 15, 2787–2794. 10.1021/acs.cgd.5b00165.

[ref28] Salvalaglio M.; Bergamaschini R.; Backofen R.; Voigt A.; Montalenti F.; Miglio L. Phase-Field Simulations of Faceted Ge/Si-Crystal Arrays, Merging into a Suspended Film. Appl. Surf. Sci. 2017, 391, 33–38. 10.1016/j.apsusc.2016.05.075.

[ref29] Albani M.; Bergamaschini R.; Salvalaglio M.; Voigt A.; Miglio L.; Montalenti F. Competition Between Kinetics and Thermodynamics During the Growth of Faceted Crystal by Phase Field Modeling. Phys. Status Solidi B 2019, 256, 1800518 10.1002/pssb.201800518.

[ref30] Masullo M.; Bergamaschini R.; Albani M.; Kreiliger T.; Mauceri M.; Crippa D.; la Via F.; Montalenti F.; von Känel H.; Miglio L. Growth and Coalescence of 3c-Sic on Si(111) Micro-Pillars by a Phase-Field Approach. Materials 2019, 12, 3223 10.3390/ma12193223. PMC6804293 31581499

[ref31] Vey S.; Voigt A. AMDiS: Adaptive Multidimensional Simulations. Comput. Visualization Sci. 2007, 10, 57–67. 10.1007/s00791-006-0048-3.

[ref32] Witkowski T.; Ling S.; Praetorius S.; Voigt A. Software Concepts and Numerical Algorithms for a Scalable Adaptive Parallel Finite Element Method. Adv. Comput. Math. 2015, 41, 1145–1177. 10.1007/s10444-015-9405-4.

[ref33] Karunasiri R. P. U.; Bruinsma R.; Rudnick J. Thin-Film Growth and the Shadow Instability. Phys. Rev. Lett. 1989, 62, 788–791. 10.1103/PhysRevLett.62.788. 10040333

[ref34] Bales G. S.; Bruinsma R.; Eklund E. a; Karunasiri R. P. U.; Rudnick J.; Zangwill A. Growth and Erosion of Thin Solid Films. Science (Washington, DC, U. S.) 1990, 249, 264–268. 10.1126/science.249.4966.264. 17750110

